# Cortical functional topography of high-frequency gamma activity relates to perceptual decision: an Intracranial study

**DOI:** 10.1371/journal.pone.0186428

**Published:** 2017-10-26

**Authors:** João Castelhano, Isabel C. Duarte, Sulaiman I. Abuhaiba, Manuel Rito, Francisco Sales, Miguel Castelo-Branco

**Affiliations:** 1 CIBIT, ICNAS, University of Coimbra, Coimbra Portugal; 2 IBILI, Faculty of Medicine, University of Coimbra, Coimbra, Portugal; 3 Epilepsy unit, CHUC, Coimbra, Portugal; University Paris 6, FRANCE

## Abstract

High-frequency activity (HFA) is believed to subserve a functional role in cognition, but these patterns are often not accessible to scalp EEG recordings. Intracranial studies provide a unique opportunity to link the all-encompassing range of high-frequency patterns with holistic perception. We tested whether the functional topography of HFAs (up to 250Hz) is related to perceptual decision-making. Human intracortical data were recorded (6 subjects; >250channels) during an ambiguous object-recognition task. We found a spatial topography of HFAs reflecting processing anterior dorsal and ventral streams, linked to decision independently of the type of processed object/stimulus category. Three distinct regional fingerprints could be identified, with lower gamma frequency patterns (<45Hz) dominating in the anterior semantic ventral object processing and dorsoventral integrating networks and evolving later, during perceptual decision phases, than early sensory posterior patterns (60-250Hz). This suggests that accurate object recognition/perceptual decision-making is related to distinct spatiotemporal signatures in the low gamma frequency range.

## Introduction

Gamma-band oscillations have been proposed to play a functional role across a wide range of cognitive domains [1–4] but their physiological underpinnings remain unclear [5–8] because a significant range of oscillatory sub-bands are not accessible to scalp EEG recordings, and even when they are, the meaning of their functional topography is difficult to unravel. This is an important challenge, because the dependence on stimulus properties, general object recognition aspects and task demands [9–11] are difficult to investigate without analyzing their precise spatial correlates. Induced gamma (in a wide range of frequencies) reflects complex processing as required in perceptual decision-making, and it is likely that such gamma becomes mixed or undetectable (for the higher ranges) at the scalp level. Therefore, the direct identification of their cortical topographic organization may help clarifying their roles [12], which can only be partially achieved using combined EEG/fMRI [13].

The seminal proposal that information transfer across neural networks is mediated by a variety of high-gamma frequencies [14,15] needs to be revisited given available evidence suggesting that this mechanism may extend up to 200Hz in many different functional domains and brain locations [16–21]. An extended role for high-gamma frequencies can more precisely be investigated through invasive recordings (ECoG) up to 500 Hz [22].

A few intracranial studies have suggested the presence of increased broad and/or narrow band gamma-oscillations during holistic perception. Lachaux et al. 2005 showed an increase of gamma activity between 50 and 150Hz in response to specific Mooney stimuli (two-tone abstract patterns depicting objects), comparing to the baseline. Hermes et al., 2014 reported a spatial distribution of increases in narrow and broadband oscillations with the broadband component being mainly located in early visual areas. Contrary to the frequently claimed stimulus specificity of the narrowband gamma oscillations, both suggested that all classes of visual stimuli induced responses in a broadband frequency range (80-200Hz) in a large portion of the fusiform gyrus and the visual cortex. Moreover, only part of the gamma activity (30-100Hz) is stimulus specific (and only in which concerns some types of stimuli: oriented gratings and only some natural images or Mooney objects). It is however possible, that in spite of the relative stimulus independence, such patterns may instead reflect general processing demands during perceptual decision-making and object recognition. Here we tested this hypothesis.

To test whether activity in the gamma-band represents particular signatures of specific processing modules, we investigated whether these patterns are organized in distinct spatial maps, as a function of the timing of perceptual decision-making. Although a specific functional role of distinct sub-bands has been suggested for different sources in the brain [23–26], this issue is complicated by the fact that these sub-bands are not always defined in the same manner in different studies. Our previous simultaneous EEG/fMRI study showed separate sources of distinct sub-bands but only for relatively low frequencies (30-45Hz; 60-75Hz) related either with perceptual processing or earlier visual processing [13]. Importantly, we found in this study that a gamma-band pattern around 40Hz emerged during successful perceptual decision-making.

There is still a lot of controversy concerning the spatial organization of those patterns of activity and their functional relevance. While some authors reported that large increases in gamma power [27] dominate in early visual areas [10,28,29], others have suggested prominent propagation of high frequency activity (HFAs) in the visual pathway [22,24,30–32]. As reviewed by Sedley and Cunningham 2013, other studies showed that broadband gamma seems to occur in various cortical areas for incongruent stimuli while narrowband patterns only occur in visual cortex. These results seem contradictory and it is not clear which are their sources and what is their relation to task dependent information processing [32–34] and in particular their link with perceptual decision-making. The relation to the timing of object recognition and decision is a critical aspect which was taken into account in our study design.

Invasive recordings are often used in the clinical context [30,35]. They provide a unique opportunity to measure brain signals at high temporal and spatial resolution [34,36–38]. This technique has been widely used to study high frequency activity [34,39,40] that cannot be uncovered by scalp EEG and their putative functional correlates have been widely debated [9,10,20,24,37]. Here we acquired intracranial data to test the following hypotheses: 1. high frequency activity is spatially organized across processing networks, i.e., their timing and functional topography reflect either early visual or late perceptual decision processes 2. their temporal patterning reflects specific object properties, or instead just a general recognition mechanism occurring during perceptual decision-making. These remain controversial hypotheses for which ECoG provides a unique opportunity to test, even with the limitations of available mathematical models for the analysis of high-density recordings at high spatio-temporal resolutions[41]. We acquired invasive data in six patients with refractory epilepsy and also extended the range of visual categories used in previous studies by including new ambiguous stimulus categories (faces, objects and scrambled stimuli that may also activate similar decision regions) [42], allowing to test general processes. Moreover, our setup involved a large sampling rate and explored the spatiotemporal dynamics of cortical activity up to 250Hz without the limitation of previously defined frequency sub-bands. Our results demonstrate a frequency dependent functional topography with lower frequencies dominating during perceptual decision-making and higher frequencies during early visual processing in more posterior regions. These maps show a strong and consistent region dependence during perceptual search and object recognition.

## Materials and methods

### Patients

We acquired invasive electrophysiology data from six epileptic patients (3 males and 3 females; see demographic and clinical details in Table 1) while they were performing a visual perceptual decision making task upon presentation of ambiguous stimuli (see details below). ECoG is a technique entailing data acquisition from intracranial electrodes of surgically implanted patients with intractable epileptic seizures. The implantation of invasive electrodes is used for further localization of seizure foci and functional mapping that is not feasible when using scalp EEG. This approach enables the extraoperative functional mapping during several days. They underwent extraoperative subdural ECoG recordings as a part of pre-surgical evaluation (data acquired between 04-Feb-2015 and 20-Jan-2017) in the epilepsy unit of Coimbra University Hospital (UMES-CHUC). The study was approved by the Ethics board of the Faculty of Medicine of the University of Coimbra and has been conducted according to the principles expressed in the Declaration of Helsinki. The ethics approval covered the testing of children and written informed consent was obtained from the participants or their parents.

**Table 1:** Demographic and clinical characteristics of the patients.

Case	Gender	Age	Seizure Type	Current Medication	Neuroanatomical Location of the intracranial EEG ^a^
**01**	M	40	Focal Dyscognitive Seizures	Carbamazepine, Valproate, Zonisamide and Levetiracetam.	Left Parieto-Temporal (Subdural Grid: 48 to 60 contacts)
**02**	F	20		N.A.	
**03**	M	39		N.A.	
**04**	M	13		N.A.	
**05**	F	35		Zosimade, Levetiracetam	
**06**	F	30		Eslicarbazepine, Clobazam, Zonisamida	Right Parieto-Temporal (Subdural Grid: 56 contacts)

ª Note that five subjects have a left grid while the 6th had the grid on the right hemisphere.

### Electrode placement

For the invasive recordings, platinum electrodes were surgically implanted along the occipito-temporal and occipito-parietal areas (1-D strips of 8 electrodes and 2-D arrays (grids) of 2x8, 4x8 and 4x5 electrodes were used). The number and placement of the electrodes was determined by the clinical needs of the patients. To avoid movement of subdural electrodes after placement they were stitched to the edge of dura mater and digital photos were taken to confirm position of the electrodes on the 3D anatomical data acquired with MRI and CT.

**Fig 1:**
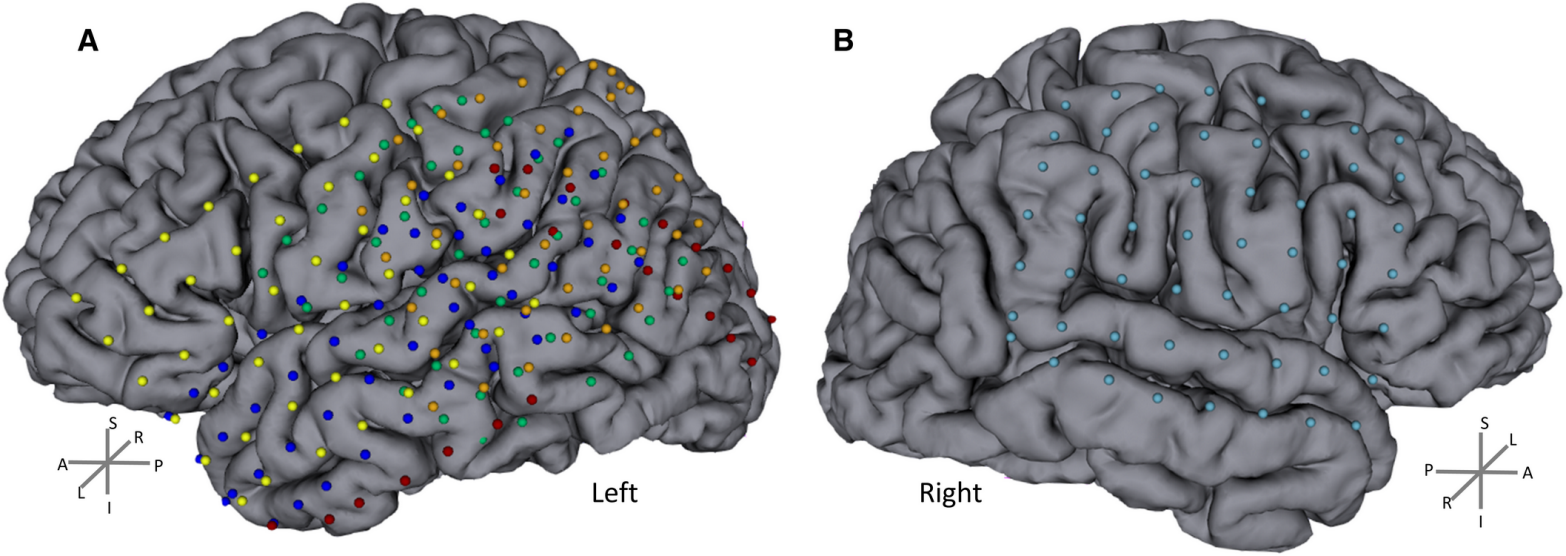
Electrode locations. A) 5 patients with left grids. B) 1 patient with grid on the right (subject 6). The individual anatomical (MRI and CT) data were co-registered to precisely localize the positions of the intracranial electrodes. Electrodes (represented as balls) were manually assigned after automatic electrode position segmentation. Each color represents one subject. Individual data were coregistered between the five subjects to obtain a single group map with the electrode locations. Note that there are matched electrode locations between subjects with an extensive coverage of both hemispheres. L/R, A/P and S/I stands for left/right, anterior/posterior and superior/inferior respectively.

Anatomical MRI, CT data and X-ray images (in which the electrodes were easily detected and localized in 3D) were acquired before and after subdural electrode placement, respectively. The individual data were then co-registered (using 3DSlicer software 4.3.0 r22408) to precisely localize the positions of the intracranial electrodes (a general registration algorithm with a linear transform was used with 6 degrees of freedom). Then, we applied a semi-automatic segmentation to extract electrode positions. The image data of the five subjects with left grids were co-registered to obtain a single map of all electrode locations (Fig 1). The data of the subject with a grid on the right hemisphere were analyzed separately.

### Task description and invasive recordings

**Fig 2:**
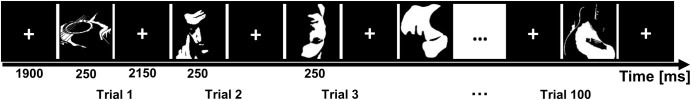
Task timeline: Mooney stimuli (ambiguous black and white shapes) of faces/inverted faces, guitars and scrambled pictures were presented for 250ms. Subjects had to detect a face (or not) and respond (button press) during the inter-stimulus interval (1900-2150ms). Stimulus order was randomized and the experiment was divided in three runs (100 trials per run) to avoid subject fatigue.

The subject was presented with four different categories of Mooney pictures. The categories included Mooney Faces, Mooney Guitars, Scrambled non-face stimuli and inverted Mooney Faces. To make the task easy to understand, for each stimulus, subjects were instructed to report if a face was present (response button 1, right hand) or not (response button 2, left hand), after stimulus offset (Fig 2). Stimuli were delivered using Presentation software (Neurobehavioral Systems). Stimulus duration was 250ms and inter-stimulus-interval varied randomly between 1900 and 2150ms. Stimulus order was randomized and the experiment was divided in three runs containing 100 stimuli per run (25 of each category). All subjects were submitted to at least 75 trials per category. Intracranial data were acquired from platinum grid electrodes at a high sampling rate (5kHz) using a 128 EEG system (SynAmps2, Compumedics, Neuroscan) used in parallel with the clinical video-EEG. No filters were applied during the recording. Data were analyzed offline.

### Data analysis

Behavioral and **reaction time** data were calculated to assess the validity of the responses to the task. All subjects were able to understand and perform the task above chance level. A Wilcoxon statistical test was applied to compare response rates per category (p = 0.05).

Then, invasive data were visually assessed by an experienced epileptologist. Electrodes including epileptic spikes or artifacts were marked as bad (~20%). After artifact correction (using independent component analysis) a common average reference (excluding bad channels) was computed to avoid distributions that are biased by the location of the reference electrode [37]. Data were split into epochs locked to the beginning of the stimuli (-500 to 1500ms) and epochs with incorrect responses were discarded (on average >70% of trials remained for further analysis). The time-window before the stimulus onset was considered as baseline (-500 – 0ms) and the baseline correction was applied.

**Fig 3:**
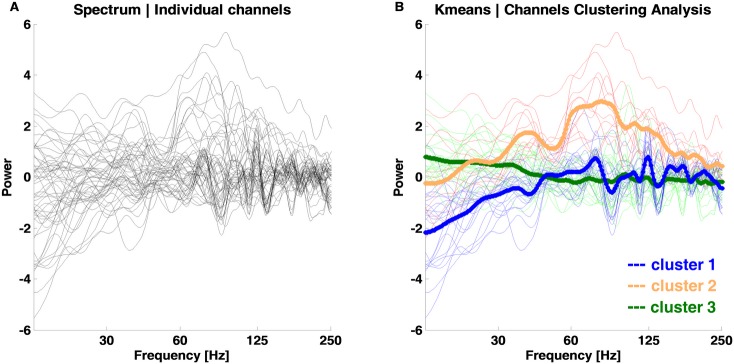
Channel clustering analysis of evoked frequency responses to faces. A) Frequency response of the 0-600ms time window per channel. Black lines: individual channels over all five subjects from the left hemisphere. B) Channel clusters as computed by the semi-automatic iterative channel clustering procedure. The default Kmeans approach with squared Euclidean distance measure was computed (best sum of distances = 6796; cluster 1 blue = 2327; cluster 2 orange = 2274; cluster 3 green = 2195). Channels belonging to the same centroid (bold lines) are marked with the same line color. The locations of these channels are plotted in the results section.

Event related spectral power (ERSP; time-frequency (TF) decomposition) analysis [43–45] was performed using the *EEGLAB* toolbox [46] and *MATLAB v8*.*1* (The Mathworks, Inc.). The wavelet parameters were: 6 cycles, 250 frequency bins, 600 time points and the analysis frequency range was set to 15 to 500Hz. A notch filter was applied (45-55Hz) to avoid potential line current artifacts. The change between post and pre-stimulus epochs was assessed. Time-frequency data per channel and subject were obtained and a semi-automatic iterative channel clustering procedure was applied based on the similarity of frequency-dependent responses to the stimuli. Briefly, we applied the k-means cluster analysis (*MATLAB R2013a*) to find the clusters of channels with a similar frequency dependent response (spectrum of the 0-600ms time window). The default squared Euclidean distance measure was used for clustering with input parameters k = 3 and 100 replicates. This clustering approach minimizes the sum, over all clusters, of the point-to-cluster centroid distances. Channels belonging to the same centroid (cluster) were concatenated per condition and analyzed together (see Fig 3 and locations in “blue”, “orange” and “green” dots in results section figures).

Statistical comparisons were performed per cluster to compare the time–frequency results with the baseline and between stimulus conditions, with the alpha level set to 0.0125. A Wilcoxon rank sum test was carried out per time and frequency bin vs. baseline values.

Additionally, we computed the spectrum as given by the time-frequency transform to assess the changes during and after stimuli presentation. The group trimmed mean (10% threshold) of the activity spectrum per time-window (stimulus ON: 25-250ms; after the stimulus/decision period: 275-600ms) was computed [47] at the single trial level. The time-window was truncated at 600ms to avoid the concomitant motor response. A margin of 25ms was excluded at the beginning and end of each time-window to avoid possible edge effects of TF computation. Next, we computed the envelope (the absolute of the Hilbert transform) of the spectrum per condition and cluster of electrodes [48]. The data of the subject with a grid on the right hemisphere were analyzed separately. A statistical analysis was then performed to obtain the frequency bins where the envelope of the power was significantly different between time-windows (Stim On vs. After Stim). The *statcond()* function from EEGLAB was applied using paired t-tests with bootstrapping (2000).

The resultant P values from the statcond function and Wilcoxon tests were corrected for multiple comparisons using a false discovery rate (FDR) approach (p = 0.0125). Furthermore, to reduce the number of type-1 errors we follow the work by [49] and corrected the statistics at the cluster level based on the time-frequency adjacency (adjacent time-frequency points that all exhibit a similar difference). The time-frequency points that reached significance after FDR correction (p<0.0125) and the cluster-based correction are marked in the TF and spectrum (envelop) plots (see results section). For the sake of clarity we call these significant TF clusters as ‘blobs’ and the similar channels are called clusters.

## Results

### Behavioral data

**Fig 4:**
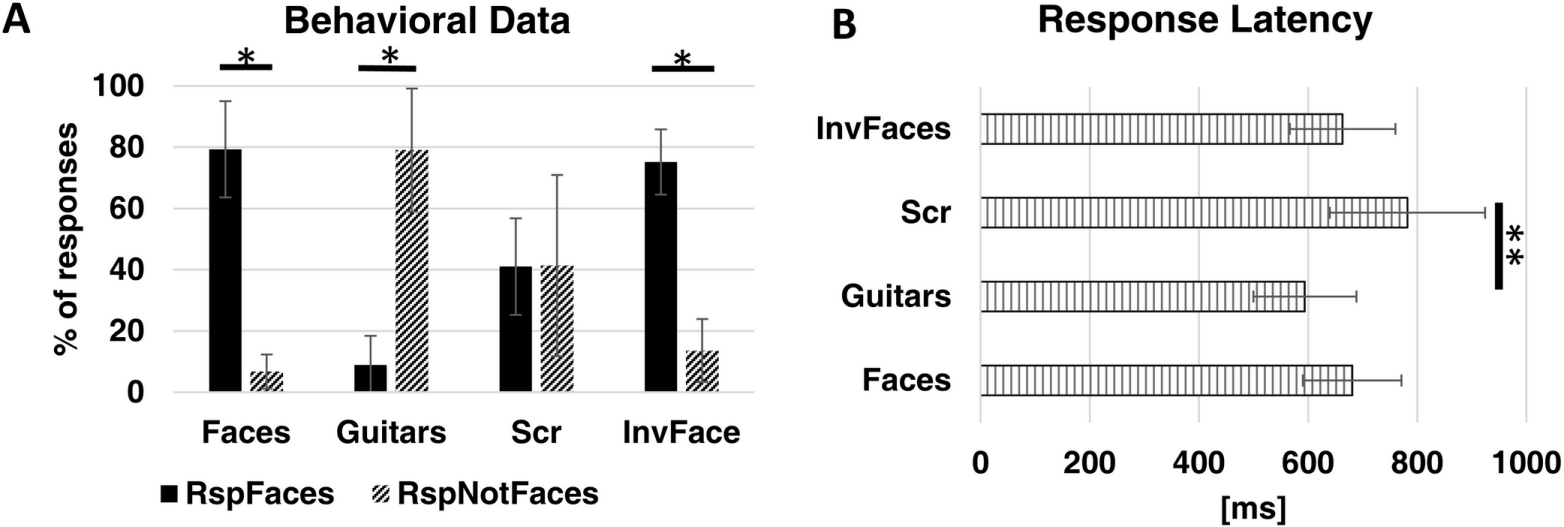
Behavioral results. A) Percentage of responses per stimuli category. *difference between responses per category with p = 0.028. B) Reaction time per stimuli condition. ** p = 0.007. Bars show the group average ± SD (N = 6).

We calculated the percentage of correct responses per category. All subjects were able to perform the task (discriminating between face and non-face objects) and could distinguish between face and non-face stimuli above the chance level. Behavioral data in Fig 4A show that ~80% of the faces/inverted faces were reported as ‘faces’ (z = 2.201, p = 0.028) while ~80% of the guitars were reported as ‘not faces’ (z = 2.201, p = 0.028). In the same line, scrambled stimuli (ambiguous) had reports of around 50% per response type (z = 0.105, p = 0.917) as was expected. Furthermore, response latencies (Fig 4B) were similar between stimulus categories and above 600ms after stimuli onset. In fact, concerning between category differences, only the Scrambled and Guitar categories differed in latency (z = -2.417, p = 0.007).

### Evidence for a spatial map of frequency distribution during perceptual decision/object recognition

We investigated high frequency activity patterns as recorded with high spatiotemporal resolution using ECoG, during ambiguous perceptual decision making. Intracranial data analysis focused on visual processing regions covered by the occipito, parietal and temporal arrays of electrodes–corresponding to early visual and the dorsal/ventral pathways, which represent an important dichotomy in high level visual processing [50,51].

**Fig 5:**
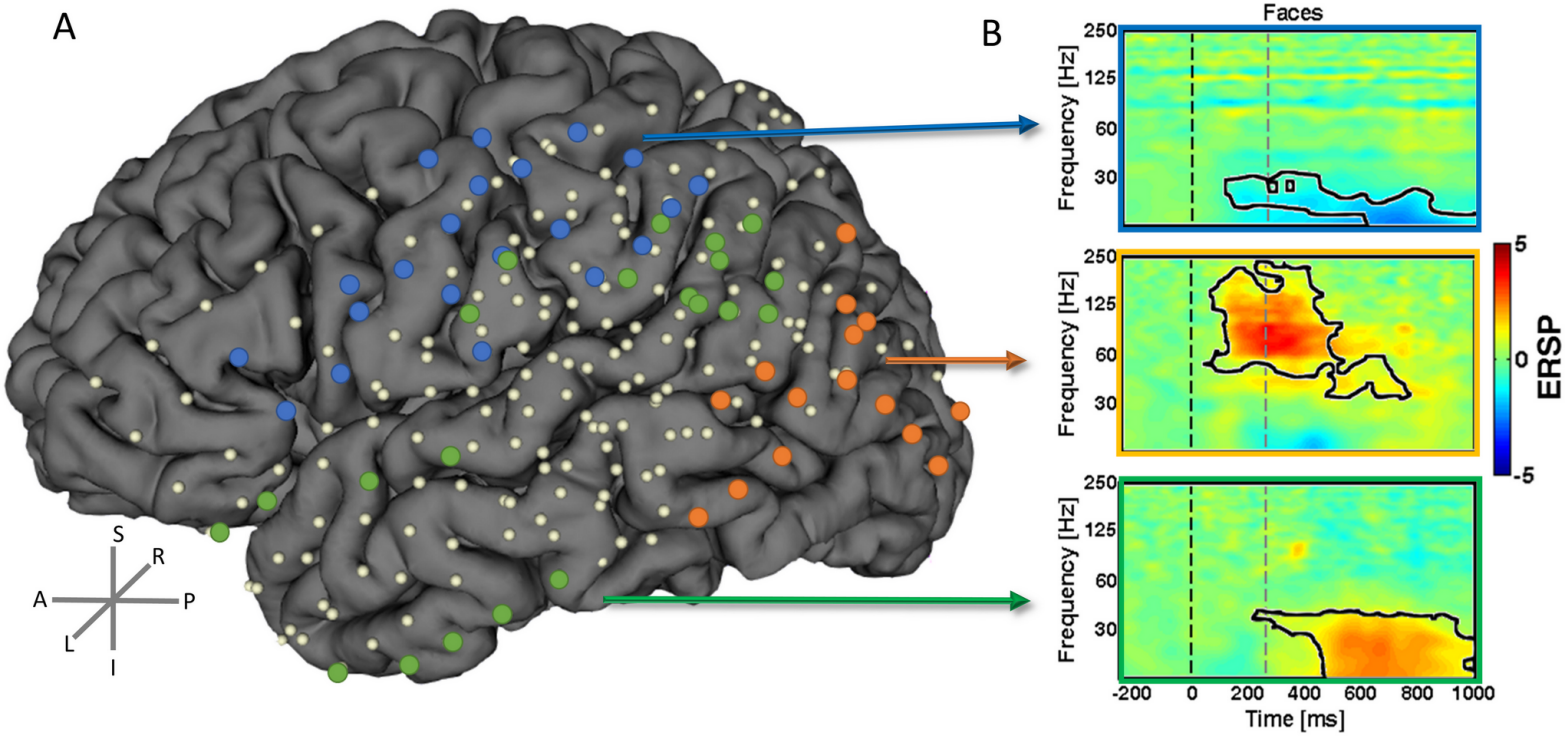
The topography of high frequency oscillations. We found distinct high frequency activity patterns during the ambiguous object recognition/perceptual decision task. The position of the electrodes per subject belonging to each of the clusters were marked as dots (in three distinct colors) in the coregistered brain. These patterns have distinct sources in the brain as represented by the correspondent dots. B) The example TF plots are a group average of all the represented electrodes of that cluster (N = 3 subjects for “blue” labels; N = 4 subjects for “orange” and “green” labels). Data (dB) are presented for the Mooney faces condition but these results could be generalized for the other conditions (see Figure A in S1 File). The black line segmented “blobs” in the plots depict the TF spectral–temporal patterns which are significant (blue z = -3.12, p<0.0017; orange, z = 3.11, p<0.0018; green, z = 3.09, p<0.0019). The dashed lines mark the start and end of stimulus.

We found distinct activity patterns occurring with distinct timing during the ambiguous visual perception decision task across distinct regions in the brain. These patterns did indeed prove to be distinct in frequency and temporal onset of significantly increased activation. These effects occurred up to the upper 250Hz boundary. We performed a semi-automatic cluster analysis (see Methods) up to this frequency range.The electrodes that showed a similar type of activity between subjects were concatenated and the average TF analysis was computed. These clusters included electrodes with increased activity over 60Hz–to 250 Hz, electrodes with increased activity below 45Hz and electrodes with decreased activity in the beta band. The topography of these high-frequency activity is shown in Fig 5A. We found a clear superior central region for the decreased beta activity as shown in electrodes labeled in blue. The early pattern of increase of higher frequencies (>60Hz–up to 250 Hz) is found in the posterior region electrodes related to early visual regions (labeled in “orange” dots). Moreover, the electrodes that represent the more anterior ventral visual processing pathways have increased later on (during perceptual decision) activation for the lower frequencies (“green” dots). These activity patterns are summarized in Fig 5B as a cluster average for the Mooney Faces category. A black line is shown to depict the TF blobs that activate significantly (FDR and cluster-based corrected for multiple comparisons) for each electrode cluster (‘blue’ -5.39<z<-3.12, 6.8E-8<p<0.0017; ‘orange’, 3.11<z<4.64, 3.39E-6<p<0.0018; ‘green’, 3.09<z<5.40, 6.8E-8<p<0.0019). These results could be generalized for the other stimulus categories and subject 6 (who was recorded in the opposite hemisphere, see Fig 1 and Figure B in S1 File). No significant differences were found between stimulus conditions after correction for multiple comparisons, suggesting that the observed topography is indeed related to general object processing mechanisms. It is important to note that lower frequencies emerge at anterior locations during late decision periods while higher frequencies are found for early visual stimulation periods at posterior sites (see next section). This is valid for all the six subjects and electrodes marked (individual graphs available upon request).

### High and low-frequency patterns show a distinct spatiotemporal topographic map during perceptual decision making

**Fig 6:**
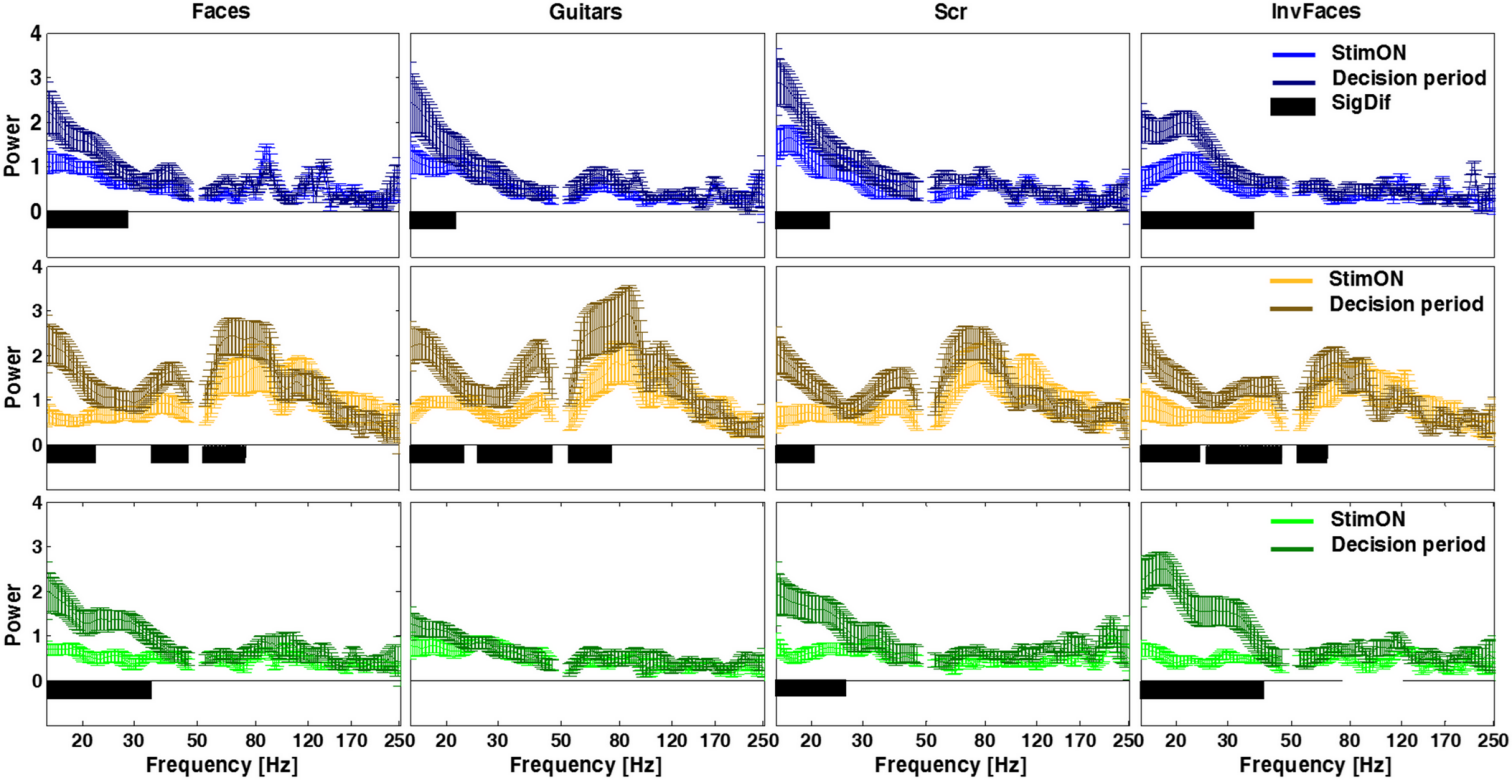
Analysis of Power per electrode cluster and condition reveals a temporal pattern of differences in power, with anterior object recognition regions showing increased decision related low frequency activity patterns. Cluster averaged power is plotted with standard deviation. Notably lower frequency band activation emerges after stimulus offset (decision period) for the more anterior electrodes (“green” and “blue”). Color lines (horizontal panels) represent the three clusters of electrodes. The more posterior channels (“orange” labels) have the main pattern increase at high gamma in contrast to the other locations. Black bars in the plots indicate the significant differences between time-windows (p<0.0065; detailed statistical values are reported in Table A in S1 file). No difference was found across stimuli conditions, suggesting that the observed regional patterns reflect a general object recognition mechanism.

To further clarify the relation of the distinct frequency band patterns with perceptual decision, the average envelope of ERSP per conditions was computed for two distinct time-windows. A statistical t-test with bootstrapping (2000) was performed (including FDR p<0.0125 and cluster-based correction for multiple comparisons) to compare the activity during and after stimulus presentation (during decision) per frequency bin. The spectrum per time-window /conditions / cluster are shown in Fig 6. We found a clear temporal pattern for the visual perception task, with power increasing at frequencies up to 250Hz in posterior electrodes (“orange”) for all stimulus conditions during the initial stimulus presentation period (1.60<t<5.91, 0.0005<p<0.0065). For the time after stimulus offset (corresponding to the decision period), the activity at posterior electrodes (“orange”) is maintained while activity for lower frequencies (<45Hz) is mainly increased at anterior electrodes (“green” and “blue”), corroborating the notion that lower frequencies are tightly linked with the decision phase at anterior ventral object recognition related regions. Note that “blue” electrodes show a decreased activity (in spite of high magnitude; 1.85<t<7.04, 0.0005<p<0.0065) while the “green” ones show increased activity (as well as high magnitude; 1.88<t<5.75, 0.0005<p<0.0065) at lower frequencies during the decision phase. The envelope of the spectrum is plotted in Fig 6 for each category and time-window showing the distinct temporal pattern of the lower frequencies in the three electrode clusters (detailed statistical output is shown in Table A in S1 File). Notably, the small standard deviation demonstrates that this holds true for all the individual subjects. The results for the subject 6 (right subdural grid; summarized in Figure B in S1 File) show a similar pattern of high frequency topography and temporal mapping for the ‘blue’ and ‘green’ clusters.

## Discussion

We were able to demonstrate a distinct topographic map of low (< 45 Hz) and high (up to 250 Hz) frequency activation patterns during a perceptual decision making task. The former were present in ventral anterior regions during the decision period and the later dominated early on during visual stimulation. These results are consistent with the notion distinct frequency bands have distinct sources and functional significance [13,27,37] and establish that they may be organized in spatiotemporal maps [23] during perceptual decision-making and object recognition.

ECoG has been widely use to study high frequency oscillations [39,40]. Here we explicitly tested whether hierarchical visual processing streams relate differently to low and high temporal frequency patterns. To address this issue we took advantage of the opportunity to perform ECoG mapping in six patients with refractory epilepsy and explored the spatiotemporal dynamics of cortical high gamma-band activity during holistic visual perceptual decision-making. We used Mooney stimuli which are known to generate a state of perceptual ambiguity during object recognition [42].

### Distinct spatial and temporal maps of high frequency activity

Previous reports suggested a functional dissociation between low and high gamma sub-bands [5,28,52,53] with distinct sources and cognitive functions [13,23,34]. Lachaux et al. 2005 showed an increase of gamma oscillations between 50 and 150Hz in response to particular Mooney stimuli, as comparing to the baseline. All classes of stimuli activated a wide portion of the fusiform gyrus and the visual cortex. Castelhano et al., 2014 had suggested, using EEG/fMRI, that frequency patterns < 45 Hz were related to perceptual decision-making, which could now explicitly test at the ECoG level.

We now provide evidence for topographic organization of visual sensory and perceptual processing and establish that these are related to a general object processing mechanism. We used a broad range of new ambiguous stimulus categories (faces, objects and scrambled stimuli that may also activate similar decision regions) [42] which allowed for generalization. Moreover, our setup comprised a higher sampling rate and explored the spatiotemporal dynamics of a broad range of cortical activity over an extensive part of the cortical mantle within whole hemispheres.

The frequency dependent spatial patterning can be summarized as follows: the low gamma and beta activity spatially involved mainly anterior regions (ventral and regions involved in dorsoventral integration) during perceptual-decision and object recognition while the higher frequencies (up to 250Hz) are increased at posterior areas [24] early on during sensory processing. Moreover, we show (Figure C in S1 File) that it is unlikely these patterns are due to epilepsy activity. These patterns are very variable and our results are an average of many trials time-locked to a stimulus event, thus rendering that influence almost null. Additionally note no overlap between the SOZ electrodes and the ones we reported as a source of the different clusters (see Figure C in S1 File). Our previous studies, ranging from single EEG, simultaneous EEG/fMRI in normal subjects and neurodevelopmental disorders provided evidence along these lines [7,8,26], in particular the link of decision processes with lower frequencies (<45 Hz), but could neither provide evidence for topographic clustering nor for exact sources. Given that many cognitive processes elicit augmentation of gamma activity [12,20,34,54] involving occipital, occipito-temporal and other inferior-frontal regions [9,37,47,55,56] invasive studies are valuable in pinpointing their neural underpinnings in a more precise way [34]. This is also emphasized by the need to parse the different number of possible processing nodes that are activated by specific stimulus categories (including faces and other impoverished stimuli) and cognitive processes and which modulate over a wide range of frequencies (up to 100Hz) [11,47].

Some evidence is available pointing to a distinction between sub-bands or differences in broad vs. sharp band patterns across different types of paradigms [13,23,27,37]. They appear to be functionally distinct and previous studies have also suggested that information is transmitted across neural networks in a variety of frequencies in the high-gamma range [10,37,57]. These and other studies have expanded the scientific interest on the relevant biological rhythms to very high frequency bands (>500 Hz) [19,22,58]. However, high-gamma power changes were recognized to not modulate uniformly across a wide range of frequencies (60-500Hz) and locations [22]. Moreover, reliable evidence for gamma patterning has been proven only for some stimuli (gratings or natural pictures) [10,55]. In this line, there is still ongoing controversy about the sources of gamma oscillations and mechanisms that generate them as a function of the cognitive process in given cortical loci [10,20,21,32].

Here we found a perceptual-decision related graded posterior-anterior sharpening of frequency band response amplitudes and their respective sources at the cortical surface level. We provided evidence that this occurs at distinct frequencies and as a function of the cognitive moment (visual processing or decision). In addition, we analyzed every frequency bin in an unbiased manner without any sort of selection. de Pesters et al. 2016 had suggested possible sources of distinct oscillatory patterns within the beta and gamma ranges and we now show a topographic dichotomy with defined functional significance. This is in line with previous EEG/fMRI evidence suggesting that lower frequency patterns are relevant during the decision period, irrespective of the object category. Additionally, we found a spatial map of decreased beta activity located at anterior motor areas. We thus suggest a posterior-anterior topographic pattern reflecting a processing hierarchy, from early visual procession to decision and object recognition.

This pattern of temporal and location precedence and dynamics of information processing is consistent with the known hierarchy of visual processing in the anterior dorsal/ventral streams. Given their close relationship to neuronal spiking, this ubiquity of high-frequency patterns, suggests that high-gamma may convey general mechanisms of cortical information transmission [16], namely object recognition. Our data, showing temporal dephasing of these patterns, are consistent with previous time-frequency analyses showing that high and low gamma power may be anti-correlated [27]. We postulate that this also holds true for each particular electrode location. Although these conclusions maybe limited by the limited sample size of invasive studies (six participants in our case), it suggests (with the added value of very high spatiotemporal resolution) that a distinct functional topographic map is present for different frequencies during visual perceptual decision-making. These results show that it is possible to parse the neural sources of high-gamma activity patterns during relatively complex tasks such as perceptual decision-making.

In sum, we have found a frequency dependent spatial patterning during object recognition, which is independent of the particular object category, suggesting a general processing mechanism. The perceptual search for an object and ensuing recognition seems to generate distinct spatio-temporal signatures during visual processing and decision. We thus found evidence in favor of the hypothesis that high frequency activity (up to 250 Hz) are spatially and temporally organized in three clusters (posterior and anterior ventral and dorsoventral) as a function of visual processing and perceptual demands.

To our knowledge, this is the first time that such a functional topographic map is shown in relation to accurate perceptual decision. Furthermore, we show also a time and space dependent (as a function of early visual processing vs. high level decision) patterning of high frequency gamma **activity**. The clear posterior-anterior pattern is consistent with hierarchical processing schemes from posterior to visual anterior dorsal/ventral streams and suggests that such object recognition related patterns are region and local connectivity dependent irrespective of the processed category.

## Supporting information

S1 File Figure A in S1 File. Group Average Time-frequency activity per condition. Event-related spectral power (dB) were computed in EEGLAB per subject and condition. This figure summarizes the TF results per condition and cluster of electrodes (three horizontal panels with distinct colors). The black lines mark the time-frequency blobs that are significantly different from the baseline (p<0.0065; Wincoxon Rank Sum test with FDR and cluster based correction for multiple comparisons). The detailed statistical values of the significant TF blobs are reported in Table A in S1 File. The dashed lines mark the start and end of the stimulus presentation. Figure B in S1 File. Time-frequency results for the individual subject 6 (right subdural grid). A) Semi-automatic clustering of the spectrum per channel. 2 channel clusters are identified (best sumd = 454.58). B) Time-frequency plots and source locations for the two clusters of channels. Data (dB) are a cluster average for the Mooney faces condition (similar results are found for the other conditions). The dashed lines mark the start and end of stimulus and the black line signal the TF blobs significantly different from the baseline (blue cluster p<0.000621; green cluster p<0.001165). C) The power envelope of distinct time-windows and clusters of electrodes for the faces condition (mean ± SD). Lower frequencies have higher power after stimulus offset. Horizontal black bar indicate the significant differences (blue cluster 2.455<t<8.5673, 0.005<p<0.0065; green cluster 1.594<t<6.5324, 0.0035<p<0.0085). Figure C in S1 File. Locations of the epilepsy related electrodes superimposed in the results image. The ‘red labeled’ electrodes represent the SOZ electrodes across all subjects while the others are the ones reported in our results. Note that there is no overlap because we did not include these ‘bad electrodes’ in the analysis. A normalized time-frequency activity (group average of these SOZ electrodes) locked to the Mooney faces stimuli is also shown. Table A in S1 File. Statistical results. The statistical test results are reported per condition and cluster of electrodes for the two main comparisons. * Wilcoxon rank sum comparison of TF data with the baseline. ** T-test with bootstrap (2000) comparison of the spectrum per time-window (Stim On vs. Decision Period). The max and minimum values of significant blobs are reported (FDR and cluster-based corrected p = 0.0125).
(DOC)
