# Supplementary material for: Cortical functional topography of high-frequency gamma activity relates to perceptual decision: an Intracranial study
Source: PLoS One. 2017 Oct 26;12(10):e0186428. doi: 10.1371/journal.pone.0186428 (PMC5657999; doi:10.1371/journal.pone.0186428)
Supplement: S1 File — Figure A in S1 File. Group Average Time-frequency activity per condition. Event-related spectral power (dB) were computed in EEGLAB per subject and condition. This figure summarizes the TF results per condition and cluster of electrodes (three horizontal panels with distinct colors). The black lines mark the time-frequency blobs that are significantly different from the baseline (p<0.0065; Wincoxon Rank Sum test with FDR and cluster based correction for multiple comparisons). The detailed statistical values of the significant TF blobs are reported in Table A in S1 File. The dashed lines mark the start and end of the stimulus presentation. Figure B in S1 File. Time-frequency results for the individual subject 6 (right subdural grid). A) Semi-automatic clustering of the spectrum per channel. 2 channel clusters are identified (best sumd = 454.58). B) Time-frequency plots and source locations for the two clusters of channels. Data (dB) are a cluster average for the Mooney faces condition (similar results are found for the other conditions). The dashed lines mark the start and end of stimulus and the black line signal the TF blobs significantly different from the baseline (blue cluster p<0.000621; green cluster p<0.001165). C) The power envelope of distinct time-windows and clusters of electrodes for the faces condition (mean ± SD). Lower frequencies have higher power after stimulus offset. Horizontal black bar indicate the significant differences (blue cluster 2.455<t<8.5673, 0.005<p<0.0065; green cluster 1.594<t<6.5324, 0.0035<p<0.0085). Figure C in S1 File. Locations of the epilepsy related electrodes superimposed in the results image. The ‘red labeled’ electrodes represent the SOZ electrodes across all subjects while the others are the ones reported in our results. Note that there is no overlap because we did not include these ‘bad electrodes’ in the analysis. A normalized time-frequency activity (group average of these SOZ electrodes) locked to the Mooney faces stim [file pone.0186428.s001.doc]

# Supplementary Material

#
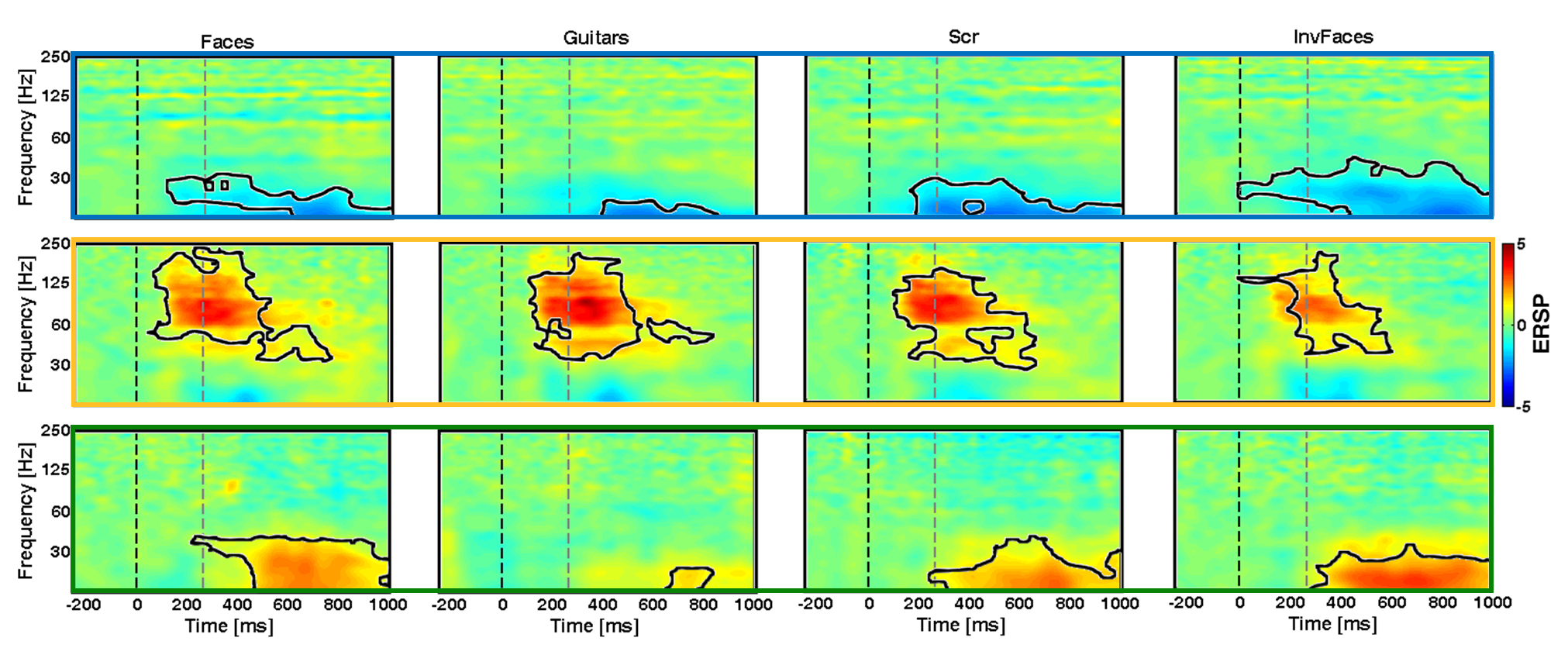


# Figure A. Group Average Time-frequency activity per condition. Event-related spectral power (dB) were computed in EEGLAB per subject and condition. This figure summarizes the TF results per condition and cluster of electrodes (three horizontal panels with distinct colors). The black lines mark the time-frequency blobs that are significantly different from the baseline (p<0.0065; Wincoxon Rank Sum test with FDR and cluster based correction for multiple comparisons). The detailed statistical values of the significant TF blobs are reported in S1 Table. The dashed lines mark the start and end of the stimulus presentation.


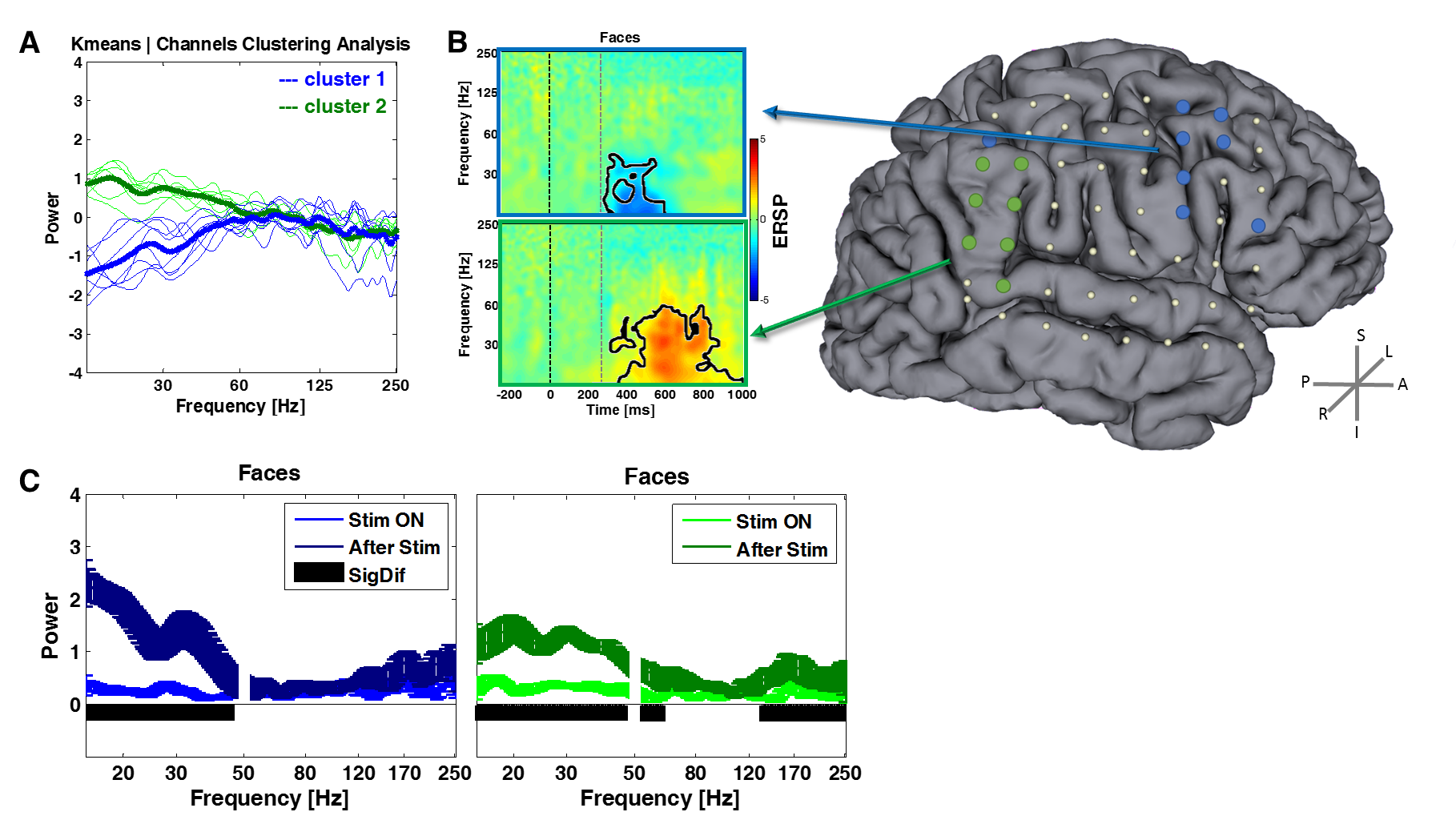


# Figure B. Time-frequency results for the individual subject 6 (right subdural grid). A) Semi-automatic clustering of the spectrum per channel. 2 channel clusters are identified (best sumd = 454.58). B) Time-frequency plots and source locations for the two clusters of channels. Data (dB) are a cluster average for the Mooney faces condition (similar results are found for the other conditions). The dashed lines mark the start and end of stimulus and the black line signal the TF blobs significantly different from the baseline (blue cluster p<0.000621; green cluster p<0.001165). C) The power envelope of distinct time-windows and clusters of electrodes for the faces condition (mean ± SD). Lower frequencies have higher power after stimulus offset. Horizontal black bar indicate the significant differences (blue cluster 2.455<t<8.5673, 0.005<p<0.0065; green cluster 1.594<t<6.5324, 0.0035<p<0.0085).

#
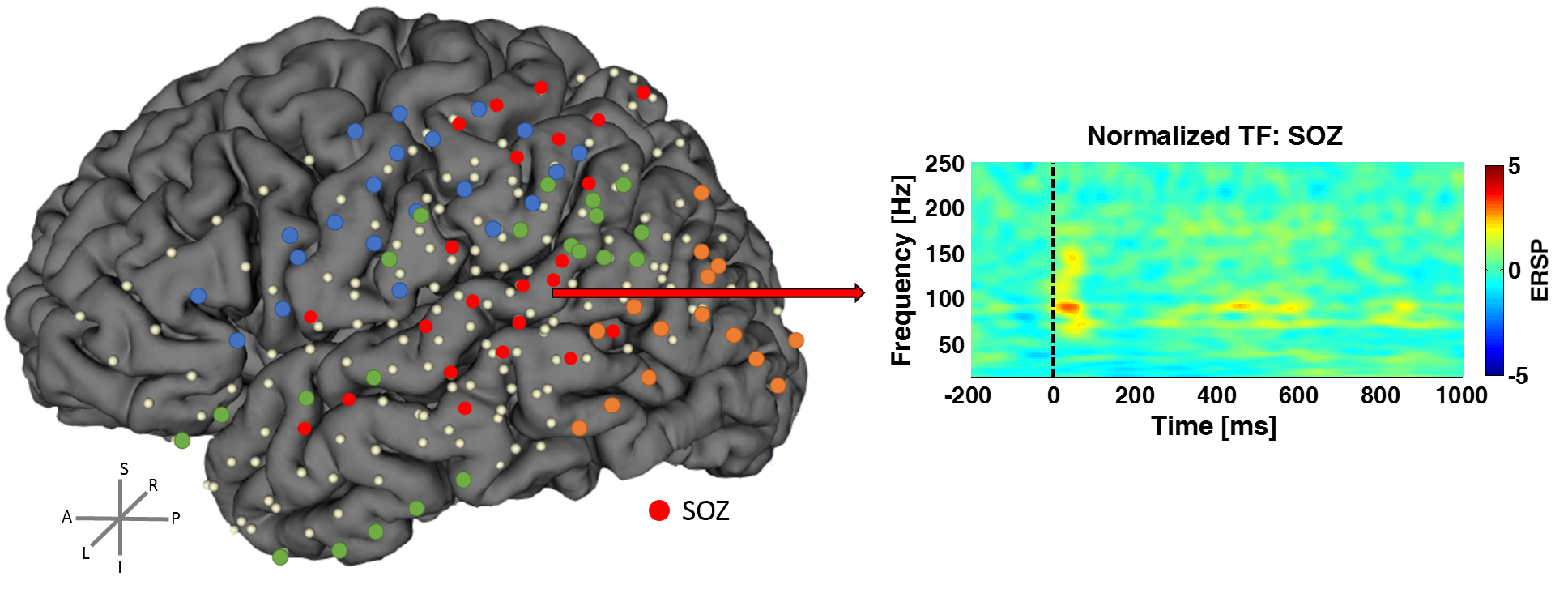


# Figure C. Locations of the epilepsy related electrodes superimposed in the results image. The ‘red labeled’ electrodes represent the SOZ electrodes across all subjects while the others are the ones reported in our results. Note that there is no overlap because we did not include these ‘bad electrodes’ in the analysis. A normalized time-frequency activity (group average of these SOZ electrodes) locked to the Mooney faces stimuli is also shown.

# Table A. Statistical results. The statistical test results are reported per condition and cluster of electrodes for the two main comparisons. * Wilcoxon rank sum comparison of TF data with the baseline. ** T-test with bootstrap (2000) comparison of the spectrum per time-window (Stim On vs. Decision Period). The max and minimum values of significant blobs are reported (FDR and cluster-based corrected p=0.0125).

| **Comparison of TF points with baseline** | **‘Orange Cluster'** | | | | **‘Green Cluster'** | | | | **‘Blue Cluster'** | | | |
| --- | --- | --- | --- | --- | --- | --- | --- | --- | --- | --- | --- | --- |
|  | **P*** | | **Z** | | **P*** | | **Z** | | **P*** | | **Z** | |
|  | **Pmax** | **Pmin** | **Zmax** | **Zmin** | **Pmax** | **Pmin** | **Zmax** | **Zmin** | **Pmax** | **Pmin** | **Zmax** | **Zmin** |
| **Faces** | 1,87E-03 | 3,39E-06 | 4,646 | 3,111 | 1,95E-03 | 6,80E-08 | 5,396 | 3,097 | 1,78E-03 | 6,80E-08 | -3,124 | -5,396 |
| **Guitars** | 1,62E-03 | 3,39E-06 | 4,646 | 3,152 | 3,05E-04 | 3,94E-07 | 5,072 | 3,611 | 7,58E-04 | 1,66E-07 | -3,368 | -5,234 |
| **Scr** | 1,40E-03 | 3,39E-06 | 4,646 | 3,194 | 2,14E-03 | 6,80E-08 | 5,396 | 3,070 | 1,12E-03 | 1,23E-07 | -3,260 | -5,288 |
| **InvFaces** | 1,05E-03 | 3,39E-06 | 4,646 | 3,277 | 1,63E-03 | 6,80E-08 | 5,396 | 3,151 | 2,34E-03 | 9,17E-08 | -3,043 | -5,342 |
|  | **P**** | | **T** | | **P**** | | **T** | | **P**** | | **T** | |
| **Comparison of Spectrum: StimOn vs Decision period** | **Pmax** | **Pmin** | **Tmax** | **Tmin** | **Pmax** | **Pmin** | **Tmax** | **Tmin** | **Pmax** | **Pmin** | **Tmax** | **Tmin** |
| **Faces** | 4,50E-03 | 5,00E-04 | 4,727 | 1,609 | 2,50E-03 | 5,00E-04 | 4,665 | 2,284 | 6,50E-03 | 5,00E-04 | 4,171 | 1,919 |
| **Guitars** | 4,50E-03 | 5,00E-04 | 5,916 | 1,694 | n.s. | n.s. | n.s. | n.s. | 2,50E-03 | 5,00E-04 | 4,481 | 1,858 |
| **Scr** | 2,50E-03 | 5,00E-04 | 3,990 | 2,161 | 4,50E-03 | 5,00E-04 | 5,314 | 1,923 | 2,50E-03 | 5,00E-04 | 5,558 | 2,153 |
| **InvFaces** | 6,50E-03 | 5,00E-04 | 3,910 | 1,829 | 6,50E-03 | 5,00E-04 | 5,751 | 1,885 | 3,50E-03 | 5,00E-04 | 7,041 | 1,933 |
